# Incorporating acute HIV infection screening, same-day diagnosis and antiretroviral treatment into routine services for key populations at sexual health clinics in Indonesia: a baseline analysis of the INTERACT prospective study

**DOI:** 10.1002/jia2.26463

**Published:** 2025-04-28

**Authors:** Irwanto Irwanto, Nurhayati H. Kawi, Hendry Luis, Dwi P. Rahmawati, Erik P. Sihotang, Pande Putu Januraga, Margareta Oktaviani, Suwarti Suwarti, Gilbert Lazarus, Evi Sukmaningrum, Evy Yunihastuti, Maartje Dijkstra, Eduard J. Sanders, F. Stephen Wignall, Keerti Gedela, Raph L. Hamers, Indonesia Intervention Study to Test & Treat People with Acute HIV Infection (INTERACT) Study Group, Irwanto, Ignatius Praptoraharjo, Arie Rahadi, Evi Sukmaningrum, Suwarti, Decy Subekti, Bachtiar Andy Mussafa, Nicolas Tarino, Mutia Rahardjani, Fitri Dewi, Soraya Weldina Ragil Dien, Margaret Oktavia, Dwi Rahmawati, Raph L. Hamers, Keerti Gedela, Pande Putu Januraga, Evy Yunihastuti, Nurhayati H. Kawi, Erik P. Sihotang, F. Stephen Wignall, Hendry Luis, F. Stephen Wignall, Godelieve de Bree, Maartje Dijkstra, Eduard Sanders, Sayem Ahmed, Christophe Fraser, Katrina Lythgoe

**Affiliations:** HIV/AIDS Research Centre, Atma Jaya Catholic University of Indonesia, Jakarta, Indonesia; Oxford University Clinical Research Unit Indonesia, Faculty of Medicine Universitas Indonesia, Jakarta; 56 Dean Street, Chelsea & Westminster Hospital, Imperial College London, UK; Center for Public Health Innovation, Udayana University, Bali; Department of Internal Medicine, Dr Cipto Mangukusumo Hospital, Faculty of Medicine, Universitas Indonesia, Jakarta; Yayasan Globalindo, Jakarta; Yayasan Bali Peduli, Bali; Amsterdam UMC, location AMC, University of Amsterdam, Amsterdam, The Netherlands; The Aurum Institute, Johannesburg, South Africa; and University of Oxford, Sir William Dunn School of Pathology, Oxford, UK; Division of Global Public Health, Brunel University London, London, UK; Big Data Institute, University of Oxford, Oxford, UK; 1 HIV/AIDS Research Centre, Atma Jaya Catholic University of Indonesia, Jakarta, Indonesia; 2 Klinik Utama Globalindo, Jakarta, Indonesia; 3 Yayasan Bali Peduli, Denpasar, Indonesia; 4 Oxford University Clinical Research Unit Indonesia, Faculty of Medicine Universitas Indonesia, Jakarta, Indonesia; 5 Center for Public Health Innovation, Udayana University, Denpasar, Indonesia; 6 Dr Cipto Mangukusumo Hospital, Faculty of Medicine Universitas Indonesia, Jakarta, Indonesia; 7 Department of Internal Medicine, Amsterdam University Medical Centers, University of Amsterdam, Amsterdam, The Netherlands; 8 The Aurum Institute, Johannesburg, South Africa; 9 Sir William Dunn School of Pathology, University of Oxford, Oxford, UK; 10 56 Dean Street Clinic, Chelsea & Westminster Hospital NHS Trust, London, UK; 11 Centre for Tropical Medicine and Global Health, Nuffield Department of Medicine, University of Oxford, Oxford, UK

**Keywords:** acute HIV infection, diagnostic algorithm, HIV testing, implementation research, Indonesia, treatment as prevention

## Abstract

**Introduction:**

Indonesia has an escalated HIV epidemic concentrated among key populations. To strengthen the care cascade, we implemented a care pathway for the screening of individuals for acute HIV infection (AHI), to achieve prompt diagnosis and antiretroviral treatment (ART) initiation, at three non-governmental sexual health clinics in Jakarta and Bali. We assessed the AHI testing uptake, yield and prevalence, and the care cascade.

**Methods:**

This is a cross-sectional baseline analysis of individuals (≥16 years) who presented for HIV testing and were consecutively enrolled (May 2023−November 2024). We used an AHI risk-score self-assessment and test algorithm comprising a fourth-generation antibody/p24 antigen rapid diagnostic test (4gRDT; Abbott Determine HIV Early Detect) and, if negative/discordant, followed by HIV-PCR (Cepheid Xpert) (either individual or pooled-sample testing). AHI was pragmatically defined as having negative/discordant RDT results with positive HIV-PCR (ISRCTN41396071).

**Results:**

Three thousand seven hundred and ninety-seven (44.0%) of 8665 individuals were screened for study eligibility, and 3689 (97.2%) were enrolled. Median age was 28 years, and 78.2% were male. Men who have sex with men (MSM) accounted for 53.3%, clients of sex workers 19.2%, persons having a sex partner living with HIV 8.9% and sex workers 4.1%. We diagnosed 229 (6.3%; 229/3662) persons with RDT-positive (chronic) HIV, and we additionally identified 13 persons with AHI—that is a diagnostic yield of 5.6% (95% CI 3.1−9.5; 13/229) overall, and 6.1% (95% CI 3.2−10.3; 12/198) among MSM. AHI prevalence was 0.38% (95% CI 0.20−0.65; 13/3429) overall, and 0.72% (95% CI 0.37−1.2; 12/1677) among MSM. The number of persons needed to test to identify one person with AHI was 264 (3429/13) overall and 140 (1677/12) among MSM. The 4gRDT’s performance to detect AHI was poor (2/13). Most participants received their HIV-PCR results on the same day (84.8%, 2907/3429) or within 24 hours (92.8%, 3182/3429). Of the 242 newly HIV-diagnosed individuals, 236 (97.5%) started ART, of whom 158 (67.0%) on the same day and 215 (91.1%) within 1 week.

**Conclusions:**

We successfully implemented prompt AHI diagnosis and treatment, and identified a high AHI prevalence among Indonesian MSM. Prioritizing access to AHI testing can create opportunities for enhanced interventions to curb the HIV epidemic among key populations.

## 1 Introduction

To end the AIDS epidemic as a public health threat, the World Health Organization (WHO) recommends that all people living with HIV (PLHIV) are started on antiretroviral treatment (ART) at the time of diagnosis, to improve individual outcomes and reduce onward HIV transmission [1, 2].

Acute HIV infection (AHI) is the time between viral acquisition and the emergence of HIV-specific antibodies (pre-seroconversion), generally accompanied by a burst of viraemia, and can be detected by HIV-PCR or p24 viral antigen test. The rate of sexual transmission during AHI has been estimated to be at least five times higher than during chronic HIV infection [3–5]. In mathematical models, AHI has been estimated to account for 10–50% of all new HIV acquisitions among men who sex with men (MSM) in Europe and the Americas [6]. At the individual level, diagnosing AHI allows prompt ART initiation, which reduces viral reservoirs [6–8] and evades irreversible damage to the host immune system [7, 8]. At the public health level, this enhances the identification of sexual partners and may reduce HIV transmission, particularly in populations with multiple sexual contacts and high HIV incidence [6, 9]. Moreover, regular AHI screening of persons using pre-exposure prophylaxis (PrEP) can enable earlier HIV diagnosis and treatment and minimize the selection of drug resistance during PrEP [10].

However, in current practice, AHI diagnosis is often missed, especially in low- and middle-income countries, thereby failing to achieve the desired full population benefits of “treat-all” strategies [6]. Current barriers to AHI diagnosis include a lack of awareness among frontline health workers, its non-specific symptoms, the inability to detect AHI with routinely used third-generation (3gRDT) antibody-based rapid diagnostic tests (RDTs), the suboptimal performance of fourth-generation antibody/p24 capsid antigen RDT (4gRDT) [6, 11] and the high costs of more sensitive HIV-PCR assays [6]. Risk-score algorithms based on symptoms and/or sexual risk behaviour have been developed to optimize the efficiency and reduce the cost of AHI screening approaches [12].

Indonesia is a socio-culturally, economically and geographically diverse, Muslim-majority, populous (275 million), middle-income country, which features stark health inequalities between regions and communities. It has one of the highest numbers of new HIV acquisitions globally, estimated at 24,000 in 2022 [13], concentrated among MSM, transgender women, and female sex workers and their sexual partners. There are substantial gaps across the HIV testing, diagnosis and treatment cascade, particularly for key populations [14–16], due to complex factors such as social stigmatization and economic and structural barriers [17, 18]. By March 2023, of an estimated 515,455 PLHIV, 85% knew their HIV status, of those 42% received ART, of those 27% had a suppressed viral load on ART [15]. Sexual health services tailored to key populations are only offered by a few private and non-government clinics. Access to oral PrEP is still limited [15, 16, 19]. Screening for AHI is not currently part of the national HIV programme. Integrating feasible, acceptable and time-sensitive AHI testing strategies into clinical settings may be an important additional intervention to realize the potential patient and population benefits of treatment-as-prevention [20–24].

The aim of the Indonesia Intervention Study to Test & Treat People with Acute HIV Infection (INTERACT) study was to assess whether implementing an AHI test-and-immediate-treat care pathway, with prompt diagnosis and ART initiation, into routine services for MSM and other key populations at sexual health clinics in Jakarta and Bali can strengthen the HIV care cascade. This paper presents a baseline analysis that assessed the AHI testing uptake, prevalence, diagnostic yield, as well as the care cascade from AHI testing to ART start.

## 2 Methods

### 2.1 Design, setting and population

INTERACT is a longitudinal study at three high-volume, non-governmental sexual health clinics in Jakarta and Bali, the provinces with the highest HIV prevalence (behind Papua). All sites provide HIV/STI prevention, testing and treatment services to key populations (70−80% MSM), collectively performing 7000–8000 HIV tests annually, at 5–10% HIV seropositivity (Figure S1). All clinic attendees who voluntarily presented for HIV testing were consecutively approached and invited to be screened for study eligibility. Individuals who elected not to be screened for study eligibility were recorded in a pre-screening log. Individuals were eligible if they: (1) were 16 years or older; (2) were not known to be living with HIV; (3) self-reported one or more risk factors of HIV acquisition (MSM; transgender woman; person who injects drugs; sex worker; client of sex workers; sexual partner of PLHIV; undisclosed); and (4) provided consent. For individuals who were not eligible or declined participation, the reason was recorded. All participants were enrolled into a care pathway for add-on AHI screening and testing at enrolment and return visits, co-designed with clinical staff and a community advisory group. Participants who were newly HIV diagnosed were counselled on their test results, offered same-day initiation of standard first-line ART (i.e. emtricitabine-tenofovir disoproxil fumarate-dolutegravir), and offered standard-of-care assisted partner notification by contract referral [25], enhanced with study-provided participant-specific referral cards that included vouchers for free partner testing. All participants who acquired HIV were followed up for 6 months as part of the study protocol (Figure S2). This paper presents a baseline analysis of all participants enrolled between May 2023 and November 2024. The study is reported as per STROBE guidelines.

### 2.2 AHI screening and test procedures

#### 2.2.1 AHI risk checker

Participants completed a self-assessment of risk factors and symptoms (“AHI risk checker”) on a mobile device (REDCap), which was slightly adjusted from a 7-item AHI risk score (1 point per item; range 0–7), based on symptoms and risk factors, that was previously validated among MSM in Amsterdam and San Diego [8, 26, 27]. The included items were (1) three or more sexual partners (adjusted from five or more, to reflect the reported median in the study population); (2) a symptomatic or laboratory-confirmed sexually transmitted infection (STI); (3) condomless receptive anal sex - each in the past 6 months; (4) weight loss; (5) fever; (6) swollen lymph nodes; or (7) oral thrush - each in the past 2 weeks. Participants were classified as high-risk if the risk score was calculated to be ≥2 (adjusted from ≥1.5, to improve the efficiency of the laboratory test algorithm).

#### 2.2.2 Laboratory test algorithm

The HIV screening test was a 4gRDT (Abbott Determine HIV Early Detect); positive or inconclusive specimens were confirmed in a serial MOH testing algorithm with 3gRDTs (Bio-line HIV1/2, or equivalent). Specimens that were either negative on 4gRDT screening test, or discordant between 4gRDT screening test and 3gRDT confirmatory testing, were additionally tested with HIV-PCR, as follows. AHI high-risk participants received a point-of-care (same-visit and individual) Xpert HIV-1 Qual assay (Cepheid) on a whole blood sample (lower limit of detection of 278 copies/ml), whereas the remnant plasma samples of all other participants underwent pooled testing using a study-specific standard operating procedure [28–30]. Briefly, up to 10 plasma samples were pooled into a volume of 1.2 ml, and tested with Xpert HIV-1 Viral Load assay (Cepheid) (lower limit of detection of 20 cps/ml) within 24 hours of collection. Reactive pools were deconstructed by testing the individual plasma samples with Xpert HIV-1 Viral Load assay and, if positive, testing a new whole blood sample with Xpert HIV-1 Qual assay (Figure S3). AHI was pragmatically defined as antibody-negative or -discordant RDTs with a positive Xpert HIV-PCR (in the absence of HIV Western blot testing). A confirmed positive HIV RDT was considered a chronic infection.

### 2.3 Data analysis

Descriptive statistics included proportions for categorical variables and median and interquartile range (IQR) for continuous variables. We used the Chi^2^ test, Fisher’s exact test or Mann−Whitney U test to compare characteristics between groups. Corresponding 95% confidence intervals were calculated using the binomial method (Clopper Pearson). Outcomes of interest were (1) number of clinic attendees who were screened for study eligibility (as a proxy for “AHI testing uptake”), calculated as the number of individuals screened for study eligibility (numerator) divided by all individuals presenting for voluntary HIV testing during the study period (excluding clients tested for antenatal care and administrative purpose only) (denominator) multiplied by 100%; (2) diagnostic yield of AHI testing, as the number of individuals with AHI (numerator) divided by individuals with antibody-positive HIV (denominator) multiplied by 100%; (3) AHI prevalence, as the number of individuals with AHI divided by individuals tested for AHI (denominator) multiplied by 100%; (4) the number needed to test (NNT) to diagnose one individual with AHI, as the number of all individuals tested for AHI (numerator) divided by individuals with AHI (denominator); (5) the sensitivity of the 4gRDT to detect AHI, as the number of individuals with AHI detected by 4gRDT (index test) divided by individuals with AHI detected by Xpert HIV-PCR (reference test) multiplied by 100%; and, lastly (6) we described the care cascade from AHI testing to ART initiation (as percentages of participants, with timelines). All analyses were performed using R version 4.3.1. A two-sided *p*<0.05 was considered significant.

### 2.4 Ethical approvals

The Atma Jaya Catholic University research ethics committee (0009R/III/PPPE.PM.10.05/10/2022) and the Oxford Tropical Research Ethics Committee (565-22) approved the study.

## 3 Results

### 3.1 Study eligibility screening and enrolment

**Figure 1:**
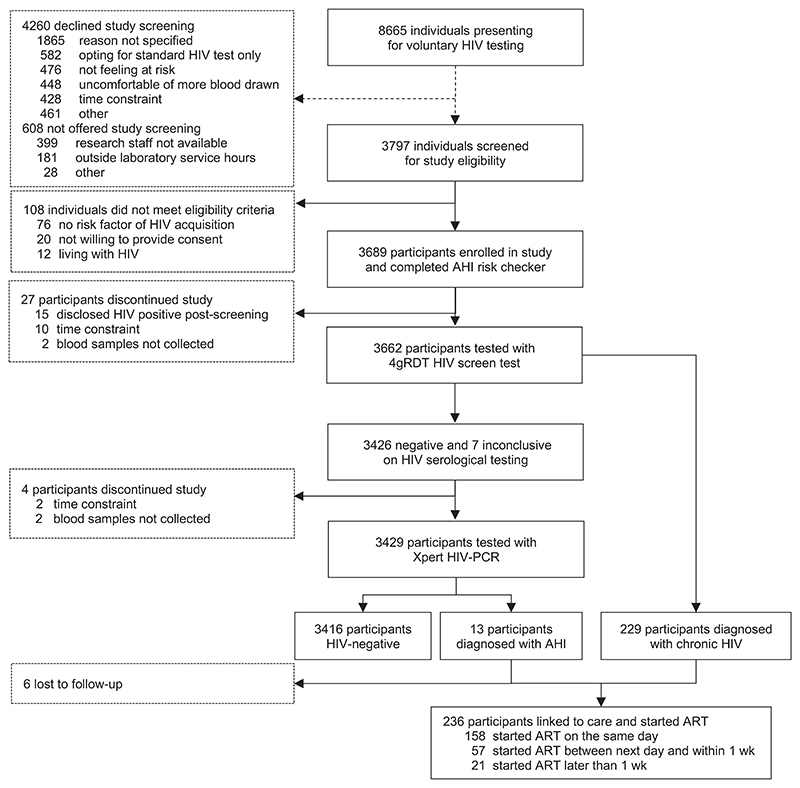
Study flow of study eligibility screening, AHI testing and ART initiation. Figure shows the aggregated data from the three study sites in Jakarta and Bali for all individuals who presented for voluntary HIV testing, were screened for study eligibility, were enrolled in the study and were tested for HIV and AHI. Acute HIV infection (AHI) was pragmatically defined as having antibody negative or discordant RDTs with a positive Xpert HIV-PCR. A confirmed positive third-generation HIV antibody rapid diagnostic test was considered chronic infection. Abbreviations: 4gRDT, fourth-generation HIV antibody/p24 antigen rapid diagnostic test; AHI, acute HIV infection; ART, antiretroviral treatment.

Of 8665 individuals presenting for standard HIV testing, 3797 (44.0%) were screened for study eligibility (Jakarta 3055/5224 [58.5%] and 742/3441 [21.6%] in Bali); whereas 4260 declined (most without providing a reason, wanting standard HIV test only or not feeling at risk) and 608 were not offered (most because research staff not available or out of laboratory service hours) (Figure 1). Of 3797 individuals screened for study eligibility, 3689 (97.2%) were enrolled (2955 in Jakarta and 734 in Bali); the reasons for not enrolling were not reporting any HIV risk (76), not willing to provide consent (20) or previously tested HIV positive (12).

### 3.2 Participant characteristics

Table 1 (Table S1 by location) summarizes the participant characteristics. Of the 3689 participants, 2884 (78.2%) identified as male, 770 (20.9%) as female, 23 (0.62%) as transgender women and 12 (0.32%) as other gender. The median age was 28 years (IQR 25–31), and 67.3% (68.8% in Jakarta and 61.4% in Bali) were below 30 years old. Most participants completed higher education (overall 70.5%, Jakarta 72.8% and Bali 61.4%) and were employed (85.3%, 85.0% and 86.4%). MSM comprised the largest key population (53.3%, 51.8% and 59.3%), followed by sex worker clients (19.2%, 21.6% and 9.7%), having a sexual partner living with HIV (8.9%, 9.1% and 8.4%), sex workers (4.1%, 3.9% and 4.6%), transgender women (0.62%, 0.3% and 1.9%), persons who inject drugs (0.41%, 0.47% and 0.14%) and those with an undisclosed risk (18.5%, 15.3% and 31.3%). 60.6% of participants (61.6% in Jakarta and 56.7% in Bali) reported to have previously taken an HIV test (Table S1).

All 3689 participants completed the AHI Risk Checker (median duration 6.2 minutes [IQR 4.9−8.2]), with 40.3% classified as high-risk (score ≥2) and median risk score 1 (IQR 1−2; range 0–7). Self-reported symptom/risk items included three or more sex partners (1708, 46.3%), condomless receptive anal sex (1173, 31.8%), STI (816, 22.1%), fever (790, 21.4%), oral thrush (439, 11.9%), enlarged lymph nodes (190, 5.2%) and weight loss (187, 5.1%) (Table 2; Table S2 by location). Condomless receptive anal sex was reported by 53.5% (1013/1893) of MSM, 7.1% (117/1659) of men who did not identify as MSM and 10.4% (71/681) of individuals not disclosing their risk. Prior PrEP use was reported by 338 (9.2%) participants (8.2% in Jakarta and 13.2% in Bali), of whom 209 (61.8%) more than a month ago. There were no location-specific differences in number of sex partners, chemsex use or condomless receptive anal sex.

### 3.3 AHI test diagnostic yield, prevalence and characteristics of individuals with AHI

**Figure 2:**
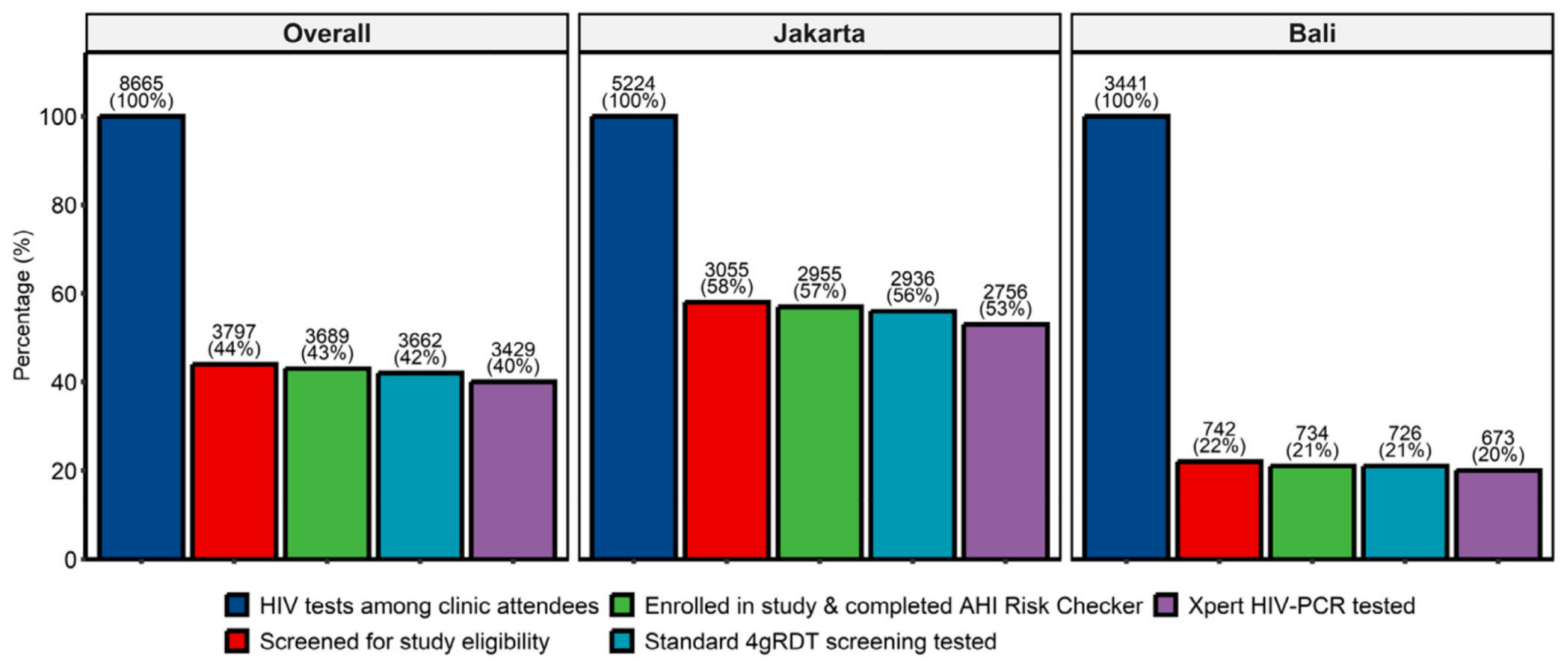
AHI testing cascade, overall and per location. Figure shows data of individuals who presented for voluntary HIV testing, who were screened for study eligibility, were enrolled in the study, and were tested for HIV and AHI, overall and per location. AHI screening comprised the AHI risk checker followed by a laboratory test algorithm of fourth-generation HIV antibody/p24 antigen rapid diagnostic screening test (4gRDT) and Xpert HIV-PCR (see Figure S3 for further details). The percentage of clinic attendees who were screened for the study (as a proxy for “AHI testing uptake”) was 44.0% (3797/8665) overall, 58.5% (3055/5224) in Jakarta and 21.6% (742/3441) in Bali. Of the 4868 (56%) individuals who were not screened for study eligibility, 4260 declined and 608 were not offered screening. Abbreviations: 4gRDT, fourth-generation HIV antibody/p24 antigen rapid diagnostic test; AHI, acute HIV infection.

Of the 3689 participants, 3662 (99.3%) underwent a 4gRDT screening test, of whom 229 (6.3%) were identified with antibody-positive (chronic) HIV and 3433 (93.7%) tested negative or inconclusive. Of those, 3429 (99.8%) underwent an Xpert HIV-PCR test, of whom 13 (0.38%) were identified with AHI (Figure 2). The additional diagnostic yield of Xpert HIV-PCR testing thus was 5.6% (95% CI 3.1−9.5; 13/229) overall, and 6.1% (95% CI 3.2−10.3; 12/198) among MSM. The NNT was 264 (3429/13) overall and 140 (1677/12) among MSM. AHI prevalence was 0.38% (95% CI 0.20−0.65; 13/3429) overall, and 0.72% (95% CI 0.37−1.2; 12/1677) among MSM (Table 3; Table S3 by location).

The 4gRDT had a low sensitivity to detect AHI (2 of 13 individuals), with reactivity to the p24 bar only without reactivity to the antibody bar.

Of the 13 participants diagnosed with AHI, the median age was 27 years (IQR 25–29; range 22–36), seven were MSM and one had an undisclosed HIV risk, and two reported recently having used PrEP (Table S4). Their median AHI risk score was 2 (IQR 2–3; range 1–4), which was statistically significantly higher than those who tested HIV negative (1, IQR 0−2; range 0–7; *p*<0.001), and comprised 11 individuals reporting condomless receptive anal sex, eight reporting fever, four reporting three or more sexual partners, four reporting oral thrush, two reporting an STI history, two reporting weight loss and one reporting enlarged lymph nodes (Table 2). Viral loads were very high (median >10^7^ cps/ml). Ten of thirteen participants with AHI started ART on the day of diagnosis. Nine individuals with AHI were offered assisted partner notification services, of whom six accepted, collectively enumerating six partners without a previous HIV diagnosis, of whom four were notified.

### 3.4 AHI test turnaround time and ART initiation

The time from starting the AHI risk checker to receiving the AHI test results was a median of 2.9 (IQR 2.5−3.8) hours for individual HIV-PCR and 5.5 hours (IQR 3.5−9.3) for pooled HIV-PCR (*p*<0.001). 84.8% (2907/3429) and 92.8% (3182/3429) received their HIV-PCR results on the same day or within 24 hours, respectively (Figure S4).

Of the 242 individuals newly diagnosed with HIV (including the 13 with AHI), 236 (97.5%) started ART, of whom 158 individuals (67.0%) started ART on the same day and 215 (91.1%) within 1 week (median 0 days, range 0–327 days), whereas 21 (11.4%) deferred ART because of a referral elsewhere (12), concurrent opportunistic infection (7) or death (2) (Figure 1). Six of the 242 (2.5%) newly HIV-diagnosed individuals (including two with AHI) did not return to the clinic and were lost to follow-up.

## 4 Discussion

This study successfully implemented an AHI self-assessment questionnaire and same-day HIV-PCR testing on individual or pooled samples at non-government sexual health clinics in Bali and Jakarta, Indonesia. This AHI-focused intervention was able to identify individuals with high viral loads and correspondingly high transmissibility, who were undetected by standard HIV tests, and initiated ART on the same day. The AHI prevalence among the MSM who participated in our study was found to be very high (0.72%), which was higher than previously reported estimates among high-risk MSM cohorts in the United States (0.19%) [31] and Amsterdam (0.32%) [8] before PrEP was rolled out. The findings were in line with the high HIV seroconversion incidence among MSM and transgender women in 2017–2020, reported in a recent retrospective analysis at sexual health clinics in Jakarta (9.4 per 100 person-years; 95% CI 7.9−11.2) and Bali (7.2 per 100 person-years; 95% CI 5.7−9.1) [32].

Several previous initiatives and studies in other settings have generated evidence for AHI-focused combination interventions. For example, in the city of Amsterdam, the implementation of a combination intervention tailored to MSM since 2014, including PrEP and an AHI test-and-immediate-treat pathway (online AHI awareness tool and point-of-care HIV-PCR testing), resulted in shortening the time between AHI diagnosis and viral suppression, detection of more persons with a recent HIV acquisition and a sharp decline of HIV incidence [33]. In a high-volume sexual health service in Bangkok, the incorporation of PCR testing in the HIV test algorithm increased the detection of AHI by 38% relative to fourth-generation immunoassays (AHI incidence of 2.2 per 100 person-years) [28], and mathematical modelling estimated that AHI detection and immediate ART initiation could reduce onward transmissions by 89% [34]. In a randomized trial in Malawi, a combination intervention of AHI screening, coupled with contract partner notification and social contact referral, increased the detection of previously undiagnosed persons and sexual partner referral per index participant compared to standard of care [35].

Indonesia faces several universal as well as context-specific challenges to achieving consistent access and continuity of HIV services, including variable political commitment to implementing comprehensive HIV programmes, community heterogeneity (e.g. geographical diversity and high mobility among key populations), structural barriers (e.g. lack of key population friendly services), high levels of stigma and discrimination against key populations and PLHIV, low levels of HIV and sexual health literacy, and lack of integrated mental health support for PLHIV, among others [16, 17, 36–38]. Integrating feasible, acceptable and time-sensitive AHI testing strategies in targeted settings in Indonesia has potential as an additional intervention to improve the care cascade for key populations and contribute to curbing HIV transmission.

We applied an existing AHI symptom/risk score that was previously validated among MSM in Amsterdam and San Diego [8, 27, 28]. However, in our study population, two of the 13 individuals diagnosed with AHI were incorrectly classified as having a low risk of AHI (based on an Amsterdam risk score <2). This finding demonstrates the need for a locally optimized and validated risk score tailored to the Indonesian MSM population, as has been developed for other settings [12, 39].

Optimal AHI test algorithms, often combining third- and/or fourth-generation serological assays with more sensitive HIV-PCR, must balance the consequences of missed diagnoses and cost, speed and ease-of-use. The diagnostic yield of the add-on Xpert HIV-PCR testing in our study population was 5.6% overall and 6.1% for MSM. For comparison, a previous meta-analysis of studies among MSM in Europe and the United States reported a pooled yield of 3.3% (95% CI 2.2−4.6%; three studies) for targeted testing among a subgroup selected based on risk behaviour and/or symptoms, as opposed to a pooled yield of 0.2% (95% CI 0.1−0.3; five studies) for universal testing [12]. By contrast, in our setting, the 4gRDT, which was performed by a laboratory technician on plasma samples, had a low sensitivity for AHI (detected only 2 of 13 individuals with AHI), which concurs with several previous reports [28, 31, 40, 41]. We, therefore, argue that in our setting AHI test algorithms should include HIV-PCR, rather than relying on 4gRDTs alone. Coupling a locally validated AHI risk score for screening, with a pooled sample testing approach and onsite HIV-PCR testing, could greatly reduce test costs while maintaining acceptable test turnaround times.

Overall, we observed a lower-than-desired “AHI testing uptake” (44% of clients were screened for the study), which corroborates existing barriers to HIV testing in this context [36–38]. The acceptance of HIV testing, and similarly of the HIV diagnosis and assisted partner notification, could be affected by fear of discrimination or disclosure of their sexual identity or HIV status, given that our study setting is one with high levels of reported societal stigma, discrimination and punitive laws against LGBTQ communities and PLHIV [36–38]. Additional factors specific to AHI testing may have played a role. Clinic attendees could have low AHI awareness and risk perception or may have been unwilling to commit to the additional time necessary for data collection. The pre-study sensitization and post-test client counselling was dependent on the clinic counselors’ communication skills, commitment and availability. Furthermore, the striking difference in “AHI testing uptake” between the sites in Jakarta (59%) and Bali (22%) may be influenced by differences between the target populations (e.g. key populations, socio-demographics, test-seeking behaviour), the accessibility of the clinic service (e.g. stigma-free services, client satisfaction, costs), community engagement models, among other factors. We are currently conducting a social science study to gain a better understanding of the context-specific reasons for declining or disengaging with AHI testing, care and assisted partner notification. We are using both provider and client perspectives to develop tailored community engagement strategies, co-designed with community stakeholders, to mitigate barriers to HIV and AHI testing and linkage to care after diagnosis [17, 18]. In May 2024, we launched a community-driven digital engagement tool called CekUpYuk.id, promoted through social media platforms, to address critical education and awareness gaps among MSM in Indonesia, which also features an AHI risk assessment tool.

There are some study limitations. First, the lower-than-desired AHI testing uptake could have introduced selection bias and influenced the AHI yield and prevalence estimates for this population. Second, we cannot rule out that our pragmatic AHI definition might have resulted in the sporadic misclassification of chronic infection as AHI. Third, the AHI risk checker relied on self-reported data, which have the potential for recall and social desirability bias [42]. Lastly, the study sites, located in urban Jakarta and Bali, were not necessarily representative of key populations in other parts of Indonesia.

## 5 Conclusions

This study is one of few initiatives in the Southeast Asian region demonstrating that AHI “test-and-immediate-treat” services can be successfully incorporated into routine HIV testing algorithms at sexual health clinics. AHI screening at scale offers the potential for enhanced interventions, including enhanced partner notification, earlier ART initiation and earlier detection of HIV breakthrough acquisitions during PrEP, all contributing to meeting Indonesia’s goal to end the HIV/AIDS epidemic. Ongoing INTERACT analyses will examine additional aspects of AHI screening, including implementation and acceptability barriers, cost-effectiveness and potential for population impact at scale. These analyses will provide critical information to design and implement sound AHI-focused testing policies for Indonesia, with relevance for other settings that have similar healthcare and structural challenges.

## Supplementary Material

Supplementary file

## Figures and Tables

**Table 1:** Participant characteristics at enrolment

	Individuals screened for study eligibility	Participants enrolled in the study		Participants who tested HIV negative	Participants who tested HIV positive	
Variable	*(N =* 3797)	(*N* = 3689)^a^	*p*-value^b^	(*N* = 3420)	(*N* = 242)	*p*-value^c^
**Age, years (median,**	28 (25–31)	28 (25–31)	0.955	28 (24–31)	28 (25–31)	0.800
**IQR)**						
16–19	106 (2.8%)	104 (2.8%)	>0.999	92 (2.7%)	10 (4.1%)	0.446
20–24	992 (26.1%)	964 (26.1%)		902 (26.4%)	60 (24.8%)	
25–29	1457 (38.4%)	1416 (38.4%)		1304 (38.1%)	99 (40.9%)	
30–34	764 (20.1%)	742 (20.1%)		694 (20.3%)	41 (16.9%)	
≥35	478 (12.6%)	463 (12.6%)		428 (12.5%)	32 (13.2%)	
**Gender identity**						
Male	2961 (78.0%)	2884 (78.2%)	0.999	2631 (76.9%)	230 (95.0%)	<0.001
Female	801 (21.1%)	770 (20.9%)		760 (22.2%)	7 (2.9%)	
Transgender	23 (0.61%)	23 (0.62%)		20 (0.58%)	3 (1.2%)	
Other^d^	12 (0.31%)	12 (0.32%)		9 (0.26%)	2 (0.83%)	
**Client status**						
First-time client	2104 (55.4%)	2031 (55.0%)	0.756	1840 (53.8%)	177 (73.1%)	<0.001
Returning client	1693 (44.6%)	1658 (45.0%)		1580 (46.2%)	65 (26.9%)	
**Education level**						
Higher education	2661 (70.5%)	2588 (70.5%)	0.998	2418 (71.1%)	151 (62.7%)	0.055
High school completed	1034 (27.4%)	1003 (27.3%)		914 (26.9%)	82 (34.0%)	
Middle school completed	64 (1.7%)	62 (1.7%)		56 (1.6%)	6 (2.5%)	
Primary school	13 (0.34%)	13 (0.35%)		11 (0.32%)	2 (0.83%)	
completed						
Primary school	4 (0.10%)	3 (0.10%)		3 (0.10%)	0 (0.0%)	
incomplete						
Not provided	21 (0.55%)	20 (0.54%)		18 (0.53%)	1 (0.41%)	
**Occupation**						
Employed	3207 (85.2%)	3119 (85.3%)	0.995	2896 (85.5%)	198 (82.2%)	0.265
Student	425 (11.3%)	412 (11.3%)		381 (11.2%)	31 (12.9%)	
Unemployed	130 (3.5%)	125 (3.4%)		112 (3.3%)	12 (5.0%)	
Not provided	35 (0.92%)	33 (0.90%)		31 (0.91%)	1 (0.41%)	
**Location**						
Jakarta	3055 (80.5%)	2955 (80.1%)	0.923	2748 (80.4%)	188 (77.7%)	0.083
Denpasar, Bali	587 (15.5%)	579 (15.7%)		534 (15.6%)	37 (15.3%)	
Ubud, Bali	155 (4.1%)	155 (4.2%)		138 (4.0%)	17 (7.0%)	
**Key population** ^e^						
Men who have sex with men	1910 (52.2%)	1893 (53.3%)	>0.999	1665 (50.6%)	210 (89.0%)	<0.001
Sex worker clients	697 (18.9%)	690 (19.2%)		662 (19.9%)	24 (10.3%)	
Sex partner living with HIV	277 (8.7%)	276 (8.9%)		248 (8.6%)	27 (14.4%)	
Sex workers	149 (4.0%)	148 (4.1%)		139 (4.1%)	6 (2.5%)	
Transgender women	23 (0.61%)	23 (0.62%)		18 (0.53%)	3 (1.2%)	
Persons who inject drugs	15 (0.40%)	15 (0.41%)		15 (0.44%)	0 (0.0%)	
Undisclosed	682 (18.0%)	681 (18.5%)		666 (19.5%)	11 (4.5%)	
**Previously HIV tested** ^f^	–	2235 (60.6%)	–	2110 (61.7%)	125 (51.7%)	0.003
**Reason for current HIV test** ^f^						
Feeling at risk	–	2233 (60.5%)	–	2047 (59.9%)	166 (68.6%)	<0.001
Retest (window period)	–	830 (22.5%)		791 (23.1%)	37 (15.3%)	
Having symptoms	–	536 (14.5%)		450 (13.2%)	81 (33.5%)	
New sexual relationship	–	529 (14.3%)		511 (14.9%)	18 (7.4%)	
Not provided	–	443 (12.0%)		421 (12.3%)	17 (7.0%)	
Getting married	–	294 (8.0%)		281 (8.2%)	12 (5.0%)	
Partner tested HIV positive	–	158 (4.3%)		139 (4.1%)	18 (7.4%)	
Partner has STI	–	64 (1.7%)		60 (1.8%)	4 (1.7%)	
Pregnant or partner	–	13 (0.35%)		12 (0.35%)	1 (0.41%)	
pregnant						

*Note*: Table shows participant’s characteristics at the three study sites combined. Data are *n* (%), unless otherwise specified. Abbreviations: IQR, interquartile range; STI, sexually transmitted infection.

a Of 3689 participants, 27 (0.7%) discontinued the study and were not tested for HIV (also refer to Figure 1).

b Individuals screened for study eligibility versus participants enrolled (Chi^2^ and Mann−Whitney U test).

c Participants who tested HIV negative versus positive (Chi^2^ and Mann−Whitney U test).

d Includes individuals who identified as non-binary or gender-fluid.

e Individuals could indicate more than one category.

f Prior HIV testing was not recorded for ineligible individuals.

**Table 2:** AHI risk score assessment and other risk factors

	All study participants	Participants who tested HIV negative	Participants who tested HIV positive		Participants who tested AHI positive	
Variable	*(N =* 3689)^a^	(*N* = 3420)	(*N* = 242)^b^	*p*-value^c^	(*N* = 13)	*p*-value^d^
**AHI risk score (median, IQR)** ^e^	1.0 (1.0–2.0)	1.0 (0.0–2.0)	2.0 (1.0–3.0)	<0.001	2.0 (2.0–3.0)	<0.001
Three or more sexual partners in the past 6 months	1708 (46.3%)	1579 (46.2%)	119 (49.2%)	0.365	4 (30.8%)	0.266
Condomless receptive anal sex in the past 6 months	1173 (31.8%)	999 (29.2%)	160 (66.1%)	<0.001	11 (84.6%)	<0.001
STI in the past 6 months	816 (22.1%)	762 (22.3%)	49 (20.3%)	0.462	2 (15.4%)	0.745
Fever in the past 2 weeks	790 (21.4%)	677 (19.8%)	108 (44.6%)	<0.001	8 (61.5%)	0.001
Oral thrush in the past 2 weeks	439 (11.9%)	388 (11.3%)	49 (20.2%)	<0.001	4 (30.8%)	0.052
Weight loss in the past 2 weeks	187 (5.1%)	138 (4.0%)	47 (19.4%)	<0.001	2 (15.4%)	0.096
Lymph nodes in the past 2 weeks	190 (5.2%)	153 (4.5%)	35 (14.5%)	<0.001	1 (7.7%)	0.450
**Number of sex partners in the past 6 months (median, IQR)**	2.0 (1.0–4.0)	2.0 (1.0–4.0)	2.0 (1.0–5.0)	0.459	2.0 (2.0–3.0)	0.660
**Anal sex in the past 3 months**	1730 (48.7%)	1535 (46.5%)	179 (78.2%)	<0.001	11 (84.6%)	0.006
Insertive/top	593 (34.3%)	560 (36.5%)	30 (16.8%)	<0.001	2 (18.2%)	0.324
Receptive/bottom	590 (34.1%)	509 (33.2%)	75 (41.9%)		6 (54.5%)	
Both insertive/top and receptive/bottom	547 (31.6%)	466 (30.4%)	74 (41.3%)		3 (27.3%)	
**Prior use of injected drugs**	15 (0.41%)	15 (0.44%)	(0.0%)	0.303	(0.0%)	>0.999
**“Chemsex” in the past 3 months** ^f^	78 (2.1%)	72 (2.1%)	6 (2.5%)	0.697	1 (7.7%)	0.244
**Group sex in the past 3 months**	202 (5.5%)	186 (5.5%)	16 (6.7%)	0.421	(0.0%)	>0.999
**Sex party in the past 3 months**	79 (2.1%)	77 (2.3%)	2 (0.83%)	0.143	(0.0%)	>0.999
**Prior use of PrEP** ^g^	338 (9.2%)	323 (9.4%)	13 (5.4%)	0.034	2 (15.4%)	0.465
More than a month ago	209 (5.7%)	198 (5.8%)	11 (4.5%)	0.044	2 (15.4%)	0.241
Less than a month ago	129 (3.5%)	125 (3.7%)	2 (0.83%)		(0.0%)	
Event-driven dosing	153 (45.3%)	146 (45.2%)	6 (46.2%)	0.978	(0.0%)	0.506
Daily dosing	183 (54.1%)	176 (54.5%)	7 (53.8%)		2 (100.0%)	

*Note*: Table summarizes the AHI risk score (range 0–7) and other risk factors for HIV acquisition in the study population, stratified by those who tested HIV negative versus positive (including AHI), and those who tested HIV negative versus AHI positive. Data are *n* (%), unless otherwise specified. Abbreviations: AHI, acute HIV infection; IQR, interquartile range; PrEP, pre-exposure prophylaxis for HIV; STI, sexually transmitted infection.

a Of 3689 participants, 27 (0.7%) discontinued the study and were not tested for HIV (also refer to Figure 1).

b Includes 229 individuals with chronic HIV (antibody positive) and 13 individuals with AHI.

c Participants who tested HIV negative versus positive (Chi^2^ and Mann−Whitney U).

d Participants who tested HIV negative versus AHI positive (Chi^2^ or Fisher exact and Mann−Whitney U).

e Adjusted from Amsterdam AHI risk score (Reference 26).

f Types of drugs used included poppers (61, 78.2%), cannabis/marijuana (10, 12.8), crystal meth (8, 10.3%), benzodiazepines and GHB (each 4, 5.1%), ecstasy/MDMA (2, 2.6%), cocaine, metamphetamine (each 1, 1.3%). Each participant can use more than one drug category.

g Mode of PrEP access included primary health centre (250, 74.0%), private clinics (46, 13.6%), ordered online (14, 4.1%), hospital (6, 1.8%) and other (22, 6.5%).

**Table 3:** Diagnostic yield of AHI testing in the study population

Variable	All participants	MSM participants
**HIV prevalence** ^a^	6.6 (5.8–7.5; 242/3662)^b^	11.2 (9.8–12.7; 210/1875)^c^
**Diagnostic yield of AHI testing**		
Any AHI risk score	5.6 (3.1–9.5; 13/229)	6.1 (3.2–10.3; 12/198)
AHI risk score ≥2	6.5 (3.3–11.3; 11/169)	6.6 (3.2–11.8; 10/151)
AHI risk score <2	3.3 (0.41–11.5; 2/60)	4.3 (0.52–14.5; 2/47)
**AHI prevalence**		
Any AHI risk score	0.38 (0.20–0.65; 13/3429)	0.72 (0.37–1.2; 12/1677)
AHI risk score ≥2	0.84 (0.42–1.5; 11/1311)	1.2 (0.60–2.3; 10/803)
AHI risk score <2	0.10 (0.01–0.34; 2/2118)	0.23 (0.03–0.82; 2/874)
**NNT to detect one individual with AHI**	264 (3429/13)	140 (1677/12)

*Note*: Data are shown as percentage (95% confidence interval; *n*/*N*) unless otherwise specified. Abbreviations: AHI, acute HIV infection; MSM, men who have sex with men; NNT, number needed to test.

a Includes participants with chronic (antibody positive) HIV (*n* = 229) and AHI (*n* = 13).

b 6.4% (95% CI 5.5−7.4; 188/2936) in Jakarta and 7.4% (95% CI 5.6−9.6; 54/726) in Bali.

c 11.4% (95% CI 9.8−13.1; 167/1468) in Jakarta and 10.6% (95% CI 7.8−14.0; 43/407) in Bali.

## Data Availability

Data are available upon reasonable request. Requests for data sharing can be made by submission of a study concept to the INTERACT Study Group for evaluation of the scientific value, relevance, design, feasibility and overlap with existing projects.
