# Supplementary material for: Incorporating acute HIV infection screening, same-day diagnosis and antiretroviral treatment into routine services for key populations at sexual health clinics in Indonesia: a baseline analysis of the INTERACT prospective study
Source: J Int AIDS Soc. Author manuscript; Available in PMC 2026 Jan 29. (PMC7618687; doi:10.1002/jia2.26463)
Supplement: Supplementary file [file EMS212193-supplement-Supplementary_file.docx]

**Table S1.** Participant characteristics at enrolment, by study location

|  | **Jakarta** | | | **Bali** | | |  |
| --- | --- | --- | --- | --- | --- | --- | --- |
| **Variable** | **Individuals screened for study eligibility**  **(N=3055)** | **Participants enrolled in the study**  **(N=2955)**^1^ | **P-value**^2^ | **Individuals screened for study eligibility**  **(N=742)** | **Participants enrolled in the study**  **(N=734)**^1^ | **P-value**^2^ | **P-value**^3^ |
| **Age, years (median, IQR)** | 28 (25-31) | 27 (25-31) | 0.920 | 28 (25-33) | 28 (25-33) | 0.979 | 0.004 |
| 16-19 | 74 (2.4%) | 72 (2.4%) | >0.999 | 32 (4.4%) | 32 (4.4%) | >0.999 | <0.001 |
| 20-24 | 820 (26.8%) | 795 (26.9%) |  | 172 (23.2%) | 169 (23.0%) |  |  |
| 25-29 | 1204 (39.4%) | 1166 (39.5%) |  | 253 (34.1%) | 250 (34.1%) |  |  |
| 30-34 | 623 (20.4%) | 602 (20.4%) |  | 141 (19.1%) | 140 (19.1%) |  |  |
| ≥35 | 334 (10.9%) | 320 (10.8%) |  | 144 (19.4%) | 143 (19.5%) |  |  |
| **Sex at birth** |  |  |  |  |  |  |  |
| Male | 2385 (78.1%) | 2315 (78.3%) | 0.798 | 638 (86.0%) | 631 (86.0%) | 0.993 | <0.001 |
| Female | 670 (21.9%) | 640 (21.7%) |  | 104 (14.0%) | 103 (14.0%) |  |  |
| **Gender identity^3^** |  |  |  |  |  |  |  |
| Male | 2348 (76.9%) | 2278 (77.1%) | 0.999 | 613 (82.6%) | 606 (82.6%) | >0.999 | <0.001 |
| Female | 687 (22.5%) | 657 (22.2%) |  | 114 (50.2%) | 113 (15.4%) |  |  |
| Transgender | 9 (0.29%) | 9 (0.30%) |  | 14 (1.9%) | 14 (1.9%) |  |  |
| Other^4^ | 11 (0.36%) | 11 (0.37%) |  | 1 (0.13%) | 1 (0.14%) |  |  |
| **Client status** |  |  |  |  |  |  |  |
| First-time client | 1664 (54.5%) | 1595 (54.0%) | 0.702 | 440 (59.2%) | 436 (59.4%) | 0.969 | 0.010 |
| Returning client | 1391 (45.5%) | 1360 (46.0%) |  | 302 (40.8%) | 298 (40.7%) |  |  |
| **Education level** |  |  |  |  |  |  |  |
| Higher education/University | 2205 (72.7%) | 2137 (72.8%) | 0.910 | 456 (61.5%) | 451 (61.4%) | >0.999 | <0.001 |
| High school completed | 797 (26.3%) | 768 (26.2%) |  | 237 (32.0%) | 235 (32.0%) |  |  |
| Middle school completed | 28 (0.9%) | 26 (0.9%) |  | 36 (4.9%) | 36 (4.9%) |  |  |
| Primary school completed | 4 (0.13%) | 4 (0.14%) |  | 9 (1.2%) | 9 (1.2%) |  |  |
| Primary school incomplete | 1 (0.03%) | 0 (0.0%) |  | 3 (0.40%) | 3 (0.41%) |  |  |
| Not provided | 20 (0.65%) | 20 (0.68%) |  | 1 (0.13%) | 0 (0.0%) |  |  |
| **Occupation** |  |  |  |  |  |  |  |
| Employed | 2568 (85.0%) | 2485 (85.0%) | 0.987 | 639 (86.2%) | 634 (86.4%) | 0.996 | <0.001 |
| Student | 386 (12.8%) | 374 (12.8%) |  | 39 (5.3%) | 38 (5.2%) |  |  |
| Unemployed | 67 (2.2%) | 63 (2.2%) |  | 63 (8.5%) | 62 (8.5%) |  |  |
| Not provided | 34 (1.1%) | 33 (1.1%) |  | 1 (0.13%) | 0 (0.0%) |  |  |
| **Key population^5^** |  |  |  |  |  |  |  |
| Men who have sex with men | 1497 (50.6%) | 1482 (51.8%) | >0.999 | 413 (59.3%) | 411 (59.3%) | >0.999 | <0.001 |
| Sex worker clients | 627 (21.1%) | 620 (21.6%) |  | 70 (9.6%) | 70 (9.7%) |  |  |
| Sex partner living with HIV | 220 (8.8%) | 220 (9.1%) |  | 57 (8.4%) | 56 (8.4%) |  |  |
| Sex workers | 116 (3.8%) | 115 (3.9%) |  | 33 (4.5%) | 33 (4.6%) |  |  |
| Transgender women | 9 (0.30%) | 9 (0.30%) |  | 14 (1.9%) | 14 (1.9%) |  |  |
| Persons who inject drugs | 14 (0.46%) | 14 (0.47%) |  | 1 (0.13%) | 1 (0.14%) |  |  |
| Undisclosed | 452 (14.8%) | 451 (15.3%) |  | 230 (31.0%) | 230 (31.3%) |  |  |
| **Previously HIV tested**^6^ | - | 1819 (61.6%) | - | - | 416 (56.7%) | - | <0.001 |
| **Reason for current HIV test**^6^ |  |  |  |  |  |  |  |
| Feeling at risk | - | 1797 (60.8%) | - | - | 436 (59.4%) | - | <0.001 |
| Retest (window period) | - | 688 (23.3%) |  | - | 142 (19.3%) |  |  |
| Having symptoms | - | 447 (15.1%) |  | - | 89 (12.1%) |  |  |
| New sexual relationship | - | 433 (14.7%) |  | - | 96 (13.1%) |  |  |
| Not provided | - | 332 (11.2%) |  | - | 111 (15.1%) |  |  |
| Getting married | - | 259 (8.8%) |  | - | 35 (4.8%) |  |  |
| Partner tested HIV-positive | - | 126 (4.3%) |  | - | 32 (4.4%) |  |  |
| Partner has STI | - | 55 (1.9%) |  | - | 9 (1.2%) |  |  |
| Pregnant or partner pregnant | - | 9 (0.30%) |  | - | 4 (0.54%) |  |  |

Table shows participant’s characteristics at enrolment per study location. Data are n (%), unless otherwise specified.

^1^ Of 3689 participants, 19 (0.6%) from Jakarta and 8 (1.1%) from Bali discontinued the study and were not tested for HIV (also refer to figure 1)

^2^ Individuals screened for study eligibility versus participants enrolled (Chi^2^ and Mann-Whitney U test)

^3^ Participants enrolled in Jakarta versus Bali (Chi^2^ and Mann-Whitney U test)

^4^ Includes individuals who identified as non-binary or gender-fluid

^5^ Individuals could indicate more than one category

^6^ Prior HIV testing was not recorded for ineligible individuals

Abbreviations: IQR, interquartile range; STI, sexually transmitted infectio

**Table S2**. AHI risk score assessment and other risk factors, by study location

| **Variable** | **Jakarta** | | | | | | **Bali** | | | | | |  | | |
| --- | --- | --- | --- | --- | --- | --- | --- | --- | --- | --- | --- | --- | --- | --- | --- |
|  | **Enrolled**  **(N=2955)** | **HIV-negative**  **(N=2748)** | **HIV-positive**  **(N=188)** | **P-value^1^** | **AHI-positive**  **(N=11)** | **P-value^2^** | **Enrolled**  **(N=734)** | **HIV-negative**  **(N=672)** | **HIV-positive**  **(N=54)** | **P-value^1^** | **AHI-positive**  **(N=2)** | **P-value^2^** | **P-value^3^** | **P-value^4^** | **P-value^5^** |
| **AHI risk score**  **(median, IQR)^6^** | 1.0  (1.0-2.0) | 1.0  (1.0-2.0) | 2.0  (2.0-3.0) | <0.001 | 2.0  (2.0-3.0) | 0.003 | 1.0  (0.0-2.0) | 1.0  (0.0-2.0) | 2.0  (1.0-3.0) | <0.001 | 3.0  (2.0-4.0) | 0.072 | <0.001 | 0.744 | 0.469 |
| Three or more sexual partners in the past 6 months | 1412  (47.8%) | 1308  (47.6%) | 96  (51.1%) | 0.357 | 2  (18.2%) | 0.051 | 296  (40.3%) | 271  (40.3%) | 23  (42.6%) | 0.744 | 2  (100%) | 0.164 | <0.001 | 0.272 | 0.077 |
| Condomless receptive anal sex in the past 6 months | 925  (31.3%) | 786  (28.6%) | 129  (68.6%) | <0.001 | 9  (81.8%) | <0.001 | 248  (33.8%) | 213  (31.7%) | 31  (57.4%) | <0.001 | 2  (100%) | 0.101 | 0.196 | 0.125 | >0.999 |
| STI in the past 6 months | 674  (22.8%) | 633  (23.0%) | 38  (20.2%) | 0.373 | 2  (18.2%) | >0.999 | 142  (19.3%) | 129  (19.2%) | 11  (20.4%) | 0.833 | 0  (0.0%) | >0.999 | 0.043 | 0.980 | >0.999 |
| Fever in the past 2 weeks | 659  (22.3%) | 571  (20.8%) | 83  (44.1%) | <0.001 | 7  (63.6%) | 0.003 | 131  (17.8%) | 106  (15.8%) | 25  (46.3%) | <0.001 | 1  (50.0%) | 0.293 | 0.008 | 0.780 | >0.999 |
| Oral thrush in the past 2 weeks | 360  (12.2%) | 322  (11.7%) | 37  (19.7%) | 0.001 | 4  (36.4%) | 0.032 | 79  (10.8%) | 66  (9.8%) | 12  (22.2%) | 0.005 | 0  (0.0%) | >0.999 | 0.288 | 0.682 | >0.999 |
| Weight loss in the past 2 weeks | 138  (4.7%) | 103  (3.7%) | 33  (17.6%) | <0.001 | 1  (9.1%) | 0.345 | 49  (6.7%) | 35  (5.2%) | 14  (25.9%) | <0.001 | 1  (50.0%) | 0.104 | 0.027 | 0.170 | 0.295 |
| Lymph nodes in the past 2 weeks | 154  (5.2%) | 128  (4.7%) | 26  (13.8%) | <0.001 | 1  (9.1%) | 0.410 | 36  (4.9%) | 25  (3.7%) | 9  (16.7%) | <0.001 | 0  (0.0%) | >0.999 | 0.736 | 0.601 | >0.999 |
| **Number of sex partners in the past 6 months**  **(median, IQR)** | 2.0  (1.0-4.0) | 2.0  (1.0-4.0) | 3.0  (1.0-5.0) | 0.398 | 2.0  (1.0-2.0) | 0.293 | 2.0  (1.0-4.0) | 2.0  (1.0-4.0) | 2.0  (1.0-4.0) | 0.915 | 4.0  (4.0-4.0) | 0.202 | 0.012 | 0.361 | 0.060 |
| **Anal sex in the past 3 months** | 1377  (48.4%) | 1218  (45.9%) | 146  (82.0%) | <0.001 | 10  (90.9%) | 0.003 | 353  (50.2%) | 317  (49.1%) | 33  (64.7%) | 0.032 | 1  (50.0%) | >0.999 | 0.399 | 0.008 | 0.295 |
| Insertive/top | 487  (35.4%) | 465  (38.2%) | 20  (13.7%) | <0.001 | 2  (20.0%) | 0.447 | 106  (30.0%) | 95  (30.0%) | 10  (30.3%) | 0.980 | 0  (0.0%) | >0.999 | 0.112 | 0.068 | >0.999 |
| Receptive/bottom | 468  (34.0%) | 398  (32.7%) | 64  (43.8%) |  | 5  (50.0%) |  | 122  (34.6%) | 111  (35.0%) | 11  (33.3%) |  | 1  (100%) |  |  |  |  |
| Both insertive/top and receptive/bottom | 422  (30.6%) | 355  (29.1%) | 62  (42.5%) |  | 3  (30.0%) |  | 125  (35.4%) | 111  (35.0%) | 12  (36.4%) |  | 0  (0.0%) |  |  |  |  |
| **Prior use of injected drugs** | 14  (0.5%) | 14  (0.5%) | 0  (0.0%) | 0.328 | 0  (0.0%) | >0.999 | 1  (0.1%) | 1  (0.1%) | 0  (0.0%) | 0.776 | 0  (0.0%) | >0.999 | 0.198 | NE | NE |
| **“Chemsex” in the past 3 months** | 58  (2.0%) | 52  (1.9%) | 6  (3.2%) | 0.216 | 1  (9.1%) | 0.192 | 20  (2.7%) | 20  (3.0%) | 0  (0.0%) | 0.199 | 2  (100%) | >0.999 | 0.200 | 0.184 | >0.999 |
| **Group sex in the past 3 months** | 69  (2.3%) | 67  (2.4%) | 2  (1.1%) | 0.233 | 0  (0.0%) | >0.999 | 10  (1.4%) | 10  (1.5%) | 0  (0.0%) | 0.366 | 0  (0.0%) | >0.999 | 0.328 | 0.444 | NE |
| **Sex party in the past 3 months** | 167  (5.7%) | 154  (5.6%) | 13  (7.0%) | 0.430 | 0  (0.0%) | >0.999 | 35  (4.8%) | 32  (4.8%) | 3  (5.6%) | 0.793 | 0  (0.0%) | >0.999 | 0.102 | 0.703 | NE |
| **Prior use of PrEP** | 241  (8.2%) | 230  (8.4%) | 10  (5.3%) | 0.140 | 1  (9.1%) | 0.931 | 97  (13.2%) | 93  (13.8%) | 3  (5.6%) | 0.084 | 1  (50.0%) | 0.140 | <0.001 | 0.946 | 0.140 |
| More than a month ago | 144  (4.9%) | 136  (4.9%) | 8  (4.3%) | 0.188 | 1  (9.1%) | 0.619 | 65  (8.9%) | 62  (9.2%) | 3  (5.6%) | 0.162 | 1  (50.0%) | 0.260 | <0.001 | 0.694 | 0.295 |
| Less than a month ago | 97  (3.3%) | 94  (3.4%) | 2  (1.1%) |  | 0  (0.0%) |  | 32  (4.4%) | 31  (4.6%) | 0  (0.0%) |  | 0  (0.0%) |  |  |  |  |
| Event-driven dosing | 101  (41.9%) | 95  (41.3%) | 5  (50.0%) | 0.848 | 0  (0.0%) | >0.999 | 52  (53.6%) | 51  (54.8%) | 1  (33.3%) | 0.462 | 0  (0.0%) | 0.457 | 0.107 | 0.612 | NE |
| Daily dosing | 139  (57.7%) | 134  (58.3%) | 5  (50.0%) |  | 1  (100%) |  | 44  (45.4%) | 42  (45.2%) | 2  (66.7%) |  | 1  (100%) |  |  |  |  |

Table summarizes the AHI risk score and other risk factors for HIV acquisition in the study population, per study location. Data are n (%) and median (IQR), unless otherwise specified.

^1^ Participants who tested HIV negative versus positive (Chi^2^ and Mann-Whitney U)

^2^ Participants who tested HIV-negative versus AHI-positive (Chi^2^ or Fisher exact and Mann-Whitney U).

^3^ Participants enrolled in Jakarta versus Bali (Chi^2^ or Fisher exact and Mann-Whitney U).

^4^ Participants who tested HIV-positive in Jakarta versus Bali (Chi^2^ or Fisher exact and Mann-Whitney U).

^5^ Participants who tested AHI-positive in Jakarta versus Bali (Chi^2^ or Fisher exact and Mann-Whitney U).

^6^ Modified from Amsterdam AHI risk score (reference 26)

**Table S3.** Diagnostic yield of AHI testing in the study population, by study location

| **Location** | **Jakarta** | | **Bali** | |
| --- | --- | --- | --- | --- |
| **Variable** | **All participants** | **MSM participants** | **All participants** | **MSM participants** |
| **HIV prevalence** | 6.4 (5.5-7.4; 188/2936) | 11.4 (9.8-13.1; 167/1468) | 7.4 (5.6-9.6; 54/726) | 10.6 (7.8-14.0; 43/407) |
| **Diagnostic yield of AHI testing** |  |  |  |  |
| Any AHI risk score | 6.2 (3.1-10.8; 11/177) | 6.4 (3.1-11.4; 10/157) | 3.8 (0.47-13.2; 2/52) | 4.9 (0.60-16.5; 2/41) |
| AHI risk score ≥2 | 6.7 (3.1-12.4; 9/134) | 6.6 (2.9-12.5; 8/122) | 5.7 (0.70-19.2; 2/35) | 6.9 (0.85-22.8; 2/29) |
| AHI risk score <2 | 4.7 (0.57-15.8; 2/43) | 5.7 (0.70-19.2; 2/35) | 0.00 (0.00-0.20; 0/17) | 0.00 (0.00-26.5; 0/12) |
| **AHI prevalence** |  |  |  |  |
| Any AHI risk score | 0.40 (0.19-0.67; 11/2756) | 0.76 (0.37-1.4; 10/1310) | 0.30 (0.04-1.1; 2/673) | 0.55 (0.06-2.0; 2/367) |
| AHI risk score ≥2 | 0.84 (0.38-1.6; 9/1074) | 1.2 (0.54-2.4; 8/645) | 0.84 (0.10-3.0; 2/237) | 1.30 (0.15-4.5; 2/158) |
| AHI risk score <2 | 0.12 (0.01-0.44; 2/1682) | 0.30 (0.04-1.1; 2/665) | 0.00 (0.00-0.84; 0/436) | 0.00 (0.00-1.8; 0/209) |
| **NNT to detect one individual with AHI** | 251 (2756/11) | 131 (1310/10) | 337 (673/2) | 184 (367/2) |

Data are shown as percentage (95% confidence interval; n/N) unless otherwise specified.

Abbreviations: AHI, acute HIV infection; MSM, men who have sex with men; NNT, number needed to test;

**Table S4.** Characteristics of the participants who were diagnosed with acute HIV infection

| **No** | **PID** | **Location** | **Date diagnosed** | **Age (yrs)** | **Client status** | **Key population** | **Prior HIV testing** | **AHI risk score^a^** | **PrEP use** | **4gRDT (R1)** | **Xpert HIV^b^** | **HIV RNA (cps/mL)** | **Assisted Partner Notification** | **ART started** |
| --- | --- | --- | --- | --- | --- | --- | --- | --- | --- | --- | --- | --- | --- | --- |
| 1 | 00062 | Bali | 29-08-23 | 29 | Returning | MSM | Yes (<6mo) | 2 (≥3 partners, CRAI) | Yes (>1 mo ago, event-driven) | INC^c^ | POC | >1x10^7 | Offered and accepted  (3 partners notified) | Yes  (same day) |
| 2 | 00074 | Jakarta | 26-05-23 | 22 | First-time | MSM | Yes (>6mo) | 2 (CRAI, fever) | Never | NEG | POC | >1x10^7 | Offered and accepted (0 partners notified) | Yes  (same day) |
| 3 | 00407 | Jakarta | 30-06-23 | 29 | First-time | MSM | Yes (>6mo) | 3 (CRAI, fever, weight loss) | Never | NEG | POC | Not done^b^ | Not offered because LTFU^f^ | Unknown^b^ |
| 4 | 00539 | Bali | 12-10-24 | 28 | First-time | MSM | Never | 4 (≥3 partners, CRAI, fever, weight loss) | Never | INC^d^ | Pool | 8.0 x10^4 | Offered but declined | Yes  (same day) |
| 5 | 00647 | Jakarta | 28-07-23 | 25 | Returning | MSM | Yes (>6mo) | 1 (fever) | Never | NEG | Pool | >1x10^7 | Not offered because LTFU^f^ | Yes (>7d) |
| 6 | 00649 | Jakarta | 28-07-23 | 25 | First-time | Undisclosed | Yes (>6mo) | 2 (STI, oral thrush) | Never | NEG | Pool | <40 | Not offered because LTFU^f^ | Unknown^b^ |
| 7 | 01100 | Jakarta | 16-09-23 | 29 | Returning | MSM | Yes (>6mo) | 3 (≥3 partners, CRAI, fever) | Never | NEG | POC | >1x10^7 | Offered but declined | Yes  (same day) |
| 8 | 01975 | Jakarta | 07-03-24 | 36 | Returning | MSM, sex worker client | Yes (>6mo) | 2 (CRAI, fever) | Yes (>1 mo ago, event-driven) | INC^c^ | POC | >1x10^7 | Offered but declined | Yes  (same day) |
| 9 | 02175 | Jakarta | 20-04-24 | 26 | Returning | MSM | Yes (>6mo) | 1 (CRAI) | Never | NEG | Pool | 2.1x10^6 | Offered and accepted  (1 partner notified) | Yes  (same day) |
| 10 | 02495 | Jakarta | 18-06-24 | 30 | Returning | MSM | Yes (<6mo) | 2 (CRAI, oral thrush) | Never | INC^d^ | Pool | 3.6x10^5 | Offered and accepted  (1 partner notified) | Yes  (same day) |
| 11 | 02539 | Jakarta | 22-06-24 | 26 | Returning | MSM | Yes (>6mo) | 3 (CRAI, fever, oral thrush) | Never | NEG | Pool | 1.0x10^5 | Offered and accepted  (1 partner notified) | Yes  (same day) |
| 12 | 02755 | Jakarta | 14-08-24 | 27 | Returning | MSM, partner living with HIV | Yes (>6mo) | 3 (CRAI, fever, lymph nodes) | Never | NEG | Pool | >1x10^7 | Offered and accepted  (0 partners notified) | Yes  (same day) |
| 13 | 02866 | Jakarta | 09-09-24 | 24 | First-time | MSM | Yes (<6mo) | 4 (≥3 partners, CRAI, STI, oral thrush) | Never | INC^e^ | Pool | 4.1x10^6 | Not offered because transfer-out | Yes  (same day) |

^a^AHI risk score (adjusted from Amsterdam score; reference 26): symptoms (fever, oral thrush, lymphadenopathy, weight loss, each in last 2 weeks) and risk factors (3 or more sex partners, condomless receptive anal intercourse, STI, each in last 6 months), each item 1 point (range 0-7).

^b^Xpert HIV-PCR was either performed as a point-of-care individual test or in a sample-pooling algorithm. See Figure S3 for the full AHI test algorithm.

^c^4gRDT HIV antigen (p24) positive and antibody-negative (R1), followed by 3gRDT 2x antibody-negative (R2+R3)

^d^4gRDT HIV antigen (p24) negative and antibody-positive (R1), followed by 3gRDT 2x antibody-negative (R2+R3)

^e^4gRDT HIV antigen (p24) positive and antibody-positive (R1), followed by 3gRDT 1x antibody-positive (R2) and 1x antibody-negative (R3)

^f^Participant did not return to clinic for confirmatory testing and was lost to follow up.

Abbreviations: 3gRDT, third-generation HIV antibody rapid diagnostic test; 4gRDT, fourth-generation HIV antibody/p24 antigen rapid diagnostic test; CRAI, condomless, receptive anal intercourse; INC, inconclusive/discordant; MSM, men who have sex with men; NEG, negative; PID, Patient ID; POC, point-of-care individual Xpert HIV-PCR test; PrEP, pre-exposure prophylaxis.

**Figure S1.** Geographic map of HIV prevalence per province


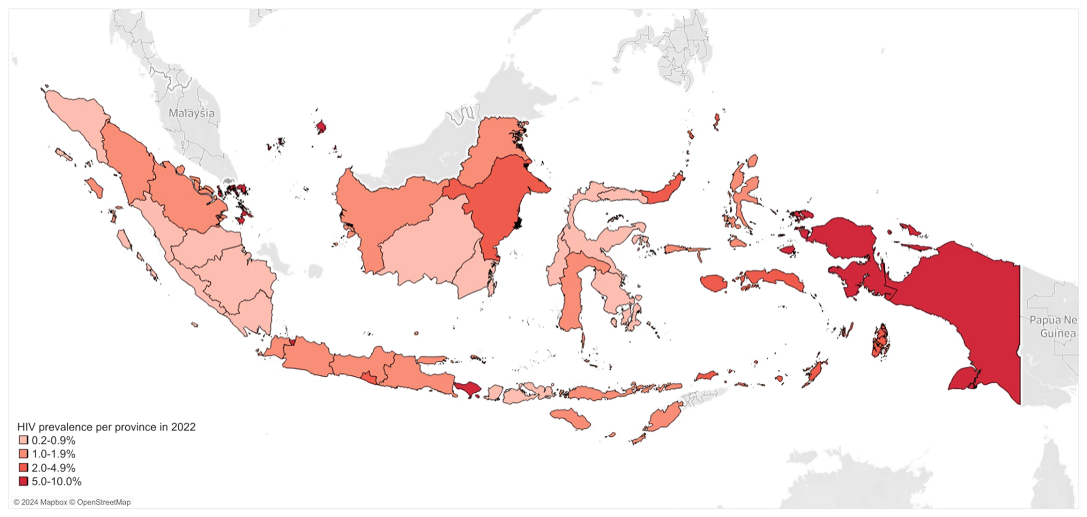

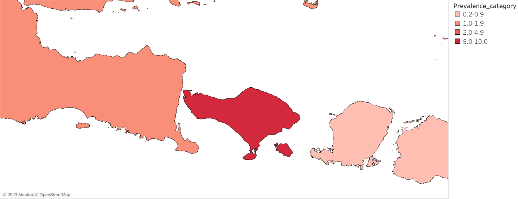

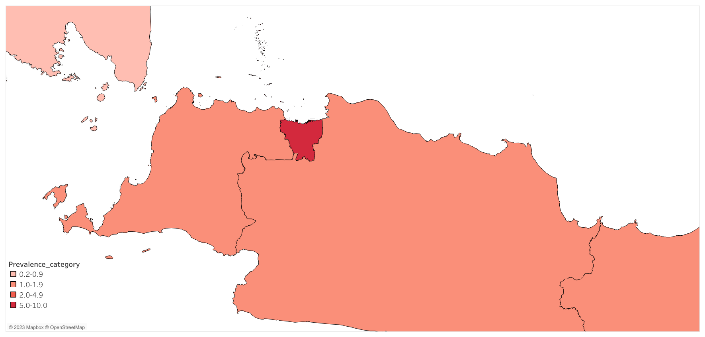


Jakarta

Bali

Figure shows a geographical map of Indonesia with the estimated HIV prevalence for each of the 34 provinces in 2022. The study sites are located in Jakarta and Bali provinces, which have the highest HIV prevalence behind Papua.

Data source: Ministry of Health (2022)

**Figure S2.** Overview of study design and procedures

S1

S2

S3

S4

Sx

**AHI Screen Visits**

*AHI testing of HIV negative participants*

**HIV Baseline and Follow-up Visits**

*Management of newly HIV diagnosed participants*

***Enrolment visit:***

Participant Information Sheet

Study Eligibility Screening

Digital consent

Maturity assessment (age 16-17 yrs)

***Baseline visit:***

Assisted partner notification

Informed consent substudy

Same-day ART start

***First and return AHI screen visits:***

AHI Risk Checker

Xpert HIV-PCR testing (individual or pooled)

REDCap data capture

T0

T3

T6

***Baseline and Follow-Up visits***

Xpert HIV-1 Viral Load testing

REDCap data capture

Sample storage (substudy)

Figure shows an overview of the INTERACT study design and procedures. After the first AHI Screening Visit at study enrolment, participants who tested AHI-negative are invited to regular return AHI Screen Visits every 3 months, or earlier if they perceive any AHI risk and/or symptoms. Successive AHI screen visits are denoted as S1, S2, S3, etc.

Participants who are newly diagnosed with HIV (including AHI) are followed up at HIV Baseline (T0) and Follow-Up Visits (T3 and T6 months).

Abbreviations: AHI, acute HIV infection; ART, antiretroviral therapy;

**Figure S3.** AHI test algorithm


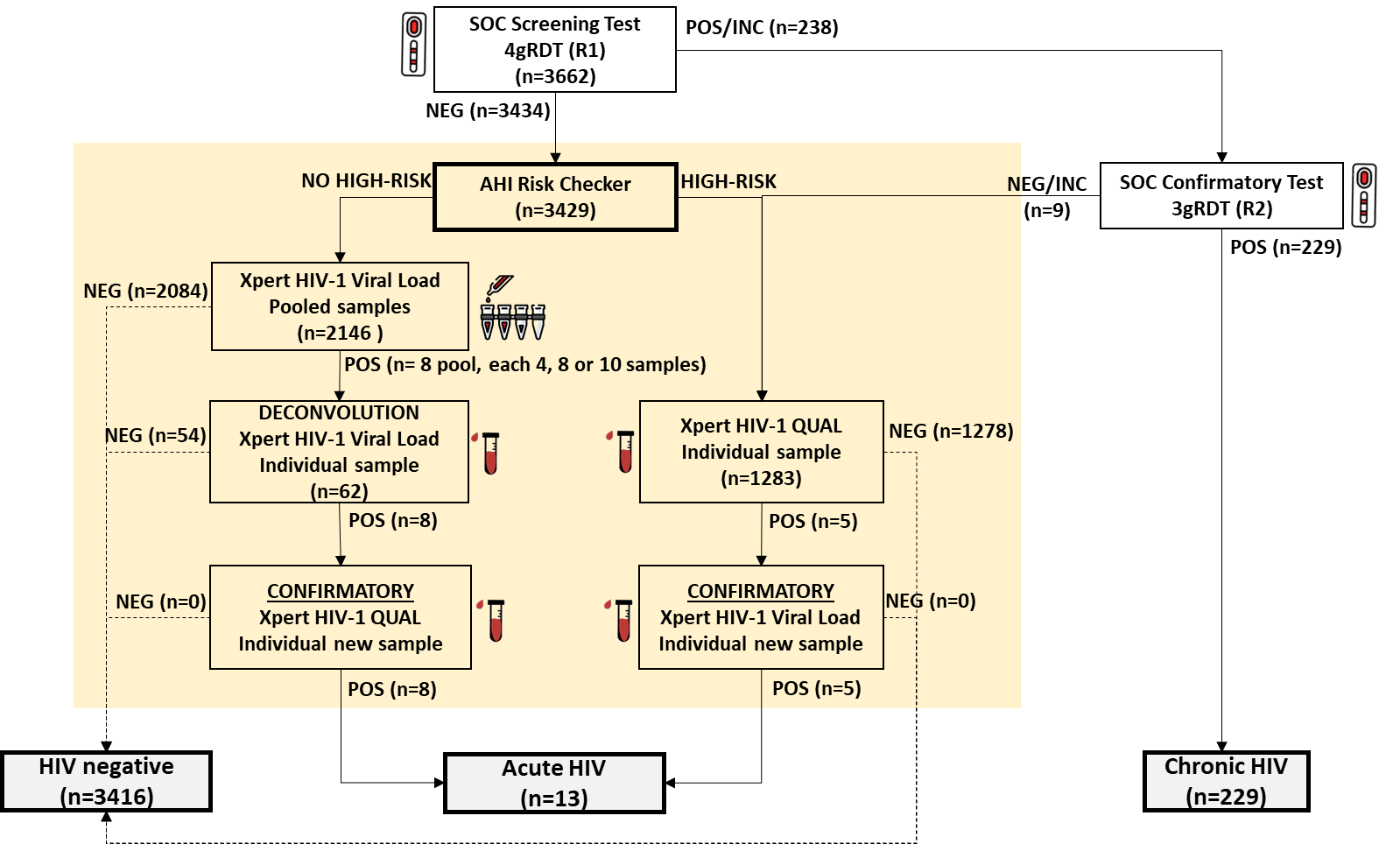


Figure shows the AHI laboratory testing algorithm used in the INTERACT study, including the numbers of samples tested and test results. The section with yellow background represent the AHI screen procedures, comprising the AHI risk checker and Xpert HIV-PCR testing.

Abbreviations: 3gRDT, third-generation HIV antibody rapid diagnostic test; 4gRDT, fourth-generation HIV antibody/p24 antigen rapid diagnostic test; AHI, acute HIV; INC, inconclusive/discordant; NEG, negative test result; POS, positive test result; SOC, standard of care;

**Figure S4.** Time to receive HIV-PCR test result for individual versus pooled testing


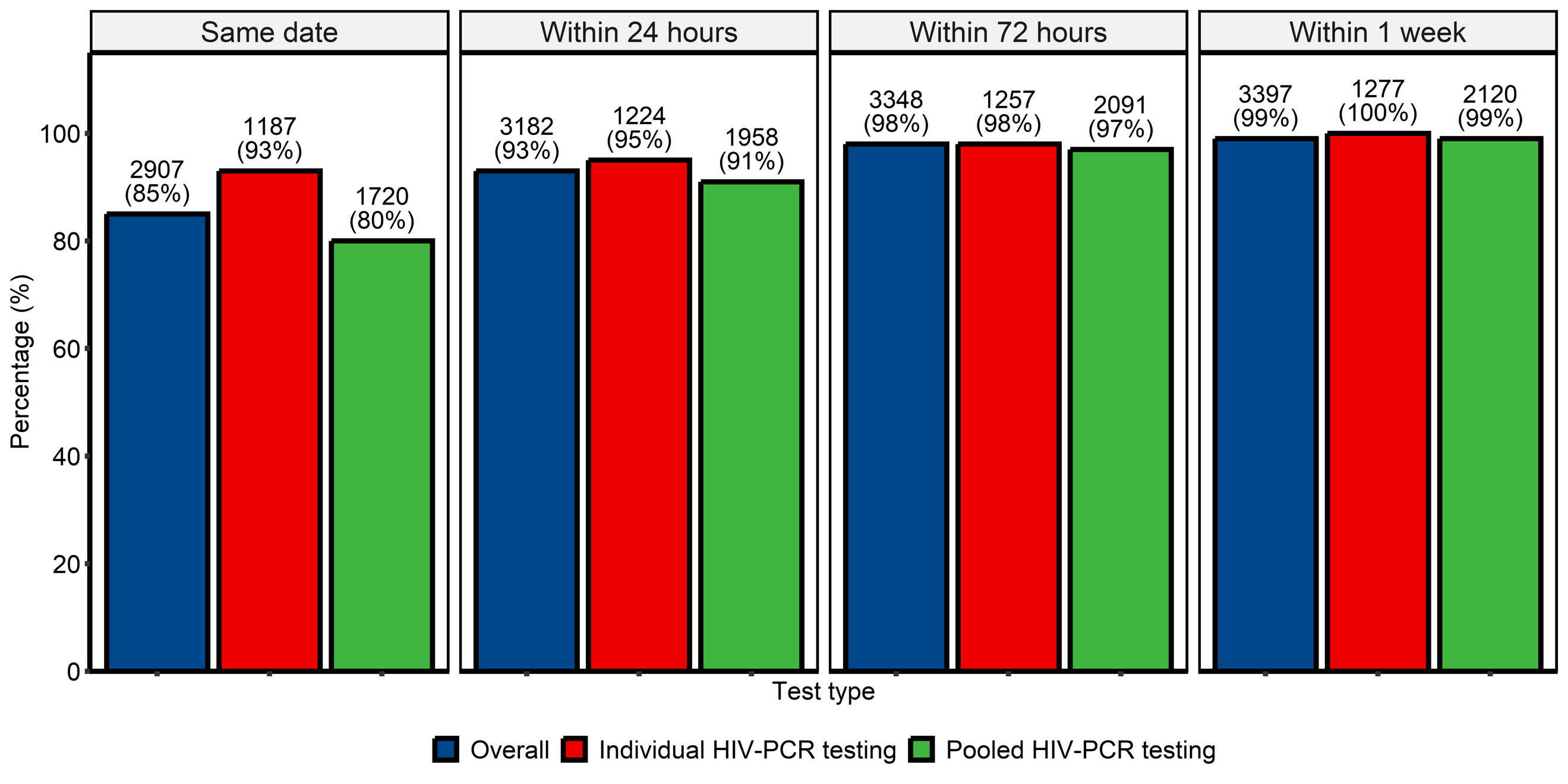


Bart chart showing the proportions of the participants tested with Xpert HIV-PCR who received the test result on the same day, within 24 hours, within 72 hours or within 1 week. The bars represent overall participants (N=3429) and divided between those who received an individual Xpert HIV-PCR test (N=1283) and those were included in the pooled HIV-PCR test approach (N=2146).

Note: 28 participants tested with pooled Xpert HIV-PCR and received the result >1 week (all tested HIV-negative).
